# Supplementary material for: Transcriptional reprogramming by mutated IRF4 in lymphoma
Source: Nat Commun. 2023 Nov 7;14:6947. doi: 10.1038/s41467-023-41954-8 (PMC10630337; doi:10.1038/s41467-023-41954-8)
Supplement: Supplementary file 1 — Supplementary Info [file 41467_2023_41954_MOESM1_ESM.pdf]

Supplementary Information Schleussner *et al.*, Transcriptional reprogramming by mutated IRF4 in lymphoma

Supplementary Figure 1

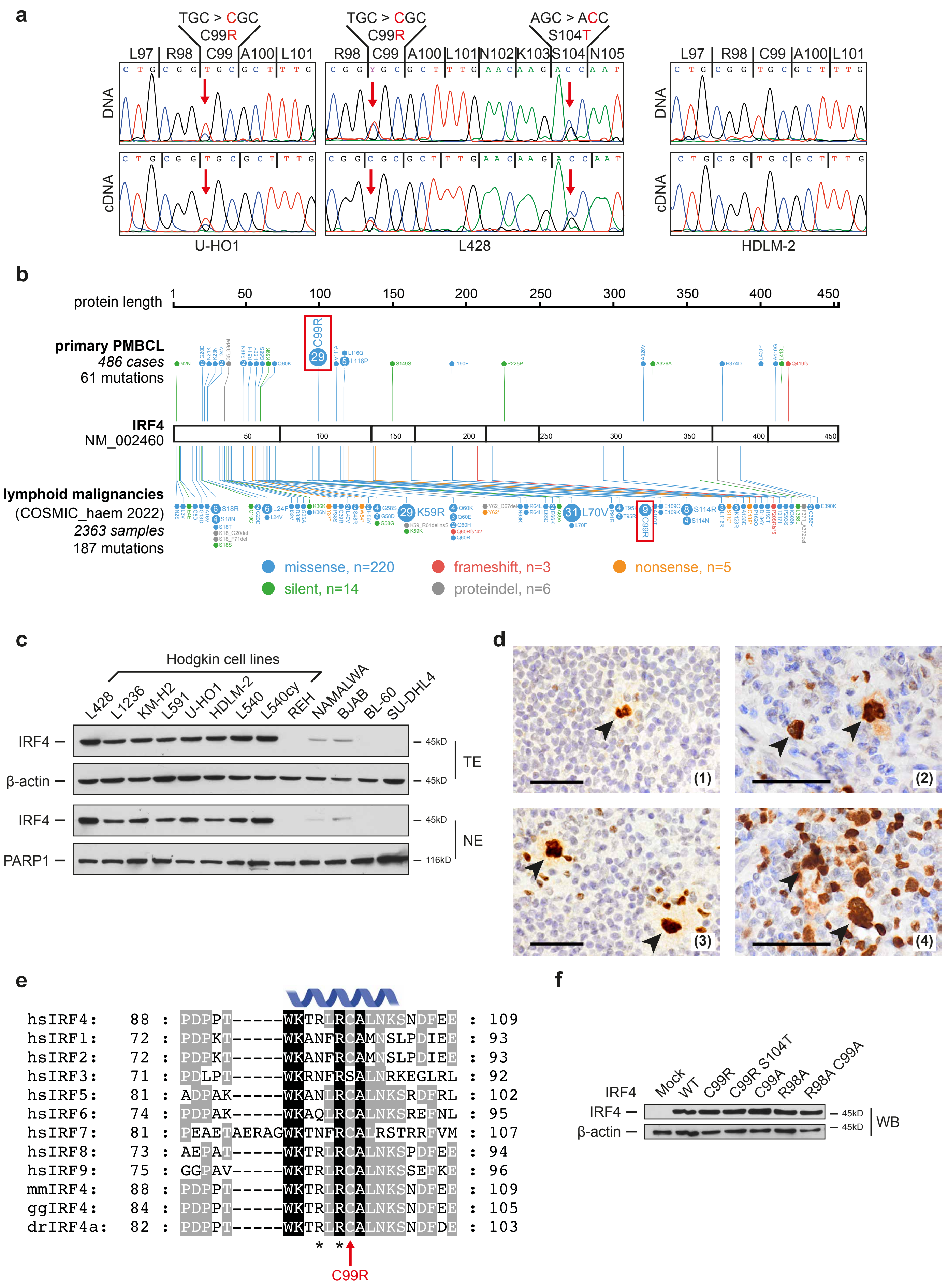

Supplementary Fig. 1. IRF4 mutations and expression in human lymphoma. (**a**) *IRF4* DNA (top panels) and cDNA (bottom panels) Sanger sequencing reads of the HRS cell lines U-HO1, harboring heterozygous IRF4-C99R, L428, harbouring heterozygous IRF4-C99R and S104T, and, as a control, HDLM-2 with IRF4-WT configuration. Red arrows indicate mutated nucleotide positions. (**b**) IRF4 mutation patterns in human lymphoma. Upper lollipop plots, IRF4-mutation frequencies in a cohort of 486 PMBCL. Lower lollipop plots, IRF4 mutation frequencies in human B cell lymphoma, numbers based on COSMIC database (https://cancer.sanger.ac.uk/cosmic; accessed on 2022/08/18) reporting 2363 samples tested. Of note, among the 9 samples harboring C99R reported in COSMIC, three samples are annotated as PMBCL and one as HL. (**c**) IRF4 immunoblotting of total extracts (TE, upper panels) or nuclear extracts (NE, lower panels) of various Hodgkin and non-Hodgkin cell lines, as indicated. Note, that HRS cell lines consistently show high IRF4 protein levels as compared to the non-Hodgkin cell lines. Data are representative of three independent experiments. (**d**) IRF4 immunohistochemistry of cHL. In total, all 90 analyzed cases (30 mixed cellularity, 30 nodular sclerosis, 30 lymphocyte-rich cHL cases) stained positive for IRF4. Representative IRF4 immunohistochemistries are shown: (1) nodular sclerosis cHL; (2) nodular sclerosis cHL; (3) lymphocyte-rich cHL; (4) mixed cellularity cHL. Magnification, 37,8X; scale bar, 50 µm. (**e**) Protein alignment of human IRF1-9 as well as of mouse (Mus musculus; mm), chicken (Gallus gallus; gg) and zebrafish (Danio rerio; dr) IRF4 sequences corresponding to AA 88-109 of human IRF4. The position of C99 is indicated by a red arrow, two adjacent highly conserved arginines are marked by asterisks. The position of the corresponding IRF4 alpha3 DNA-recognition helix is shown in blue above the sequences. (**f**) Protein expression controls related to Fig. 1b. Nuclear extracts of HEK293 cells transfected with control plasmid (Mock) or the respective IRF4 variants, as indicated, were analyzed by immunoblotting for expression of IRF4 and, as a control, β-actin. Data are representative of at least three independent experiments. Source data for Supplementary figure parts 1c and 1f are provided in the Source Data file.

Supplementary Figure 2

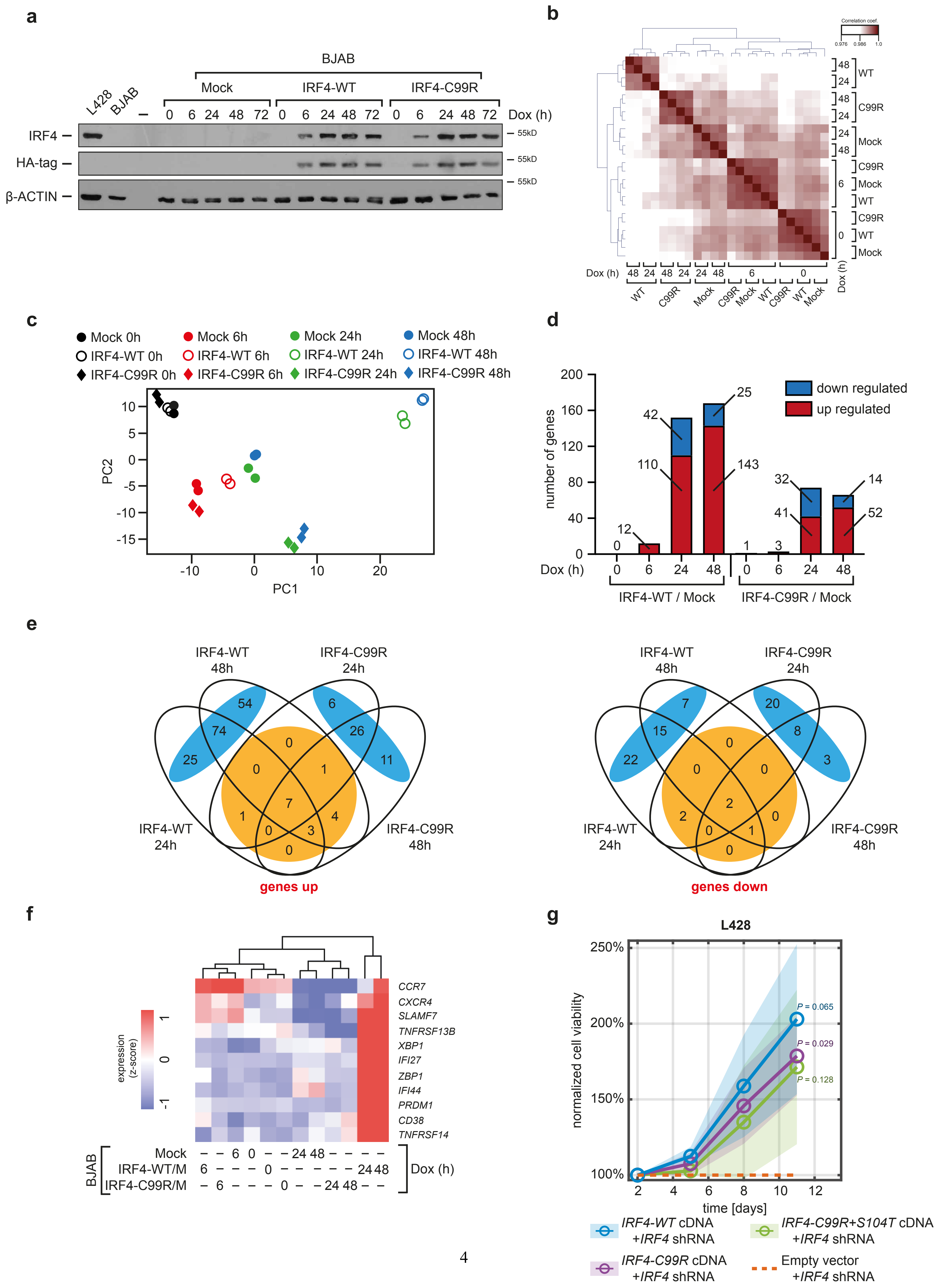

Supplementary Fig. 2. IRF4-C99R functionality: IRF4-C99R regulates less and distinct genes compared to IRF4-WT and rescues HRS cells as efficiently as IRF4-WT following IRF4 knock-down. (**a**) Mock, HA-tag-IRF4-WT and HA-tag-IRF4-C99R Tet-inducible non-Hodgkin BJAB cells were treated with doxycycline (Dox) for the indicated times. At each time, cells were analyzed by immunoblotting for IRF4 protein levels by use of antibodies to IRF4 or HA-tag. Extracts of untreated cHL L428 and non-Hodgkin BJAB cells (far left) as well as expression of β-actin were analyzed as positive or negative controls, respectively. One out of three independent experiments is shown. (**b**) Hierarchical clustering of Pearson correlation between the various transfectants, as indicated, across all expressed genes. Note, that control and respective IRF4 variants form separate clusters indicating differential gene regulation. (**c**) A two-dimension principal component plot between PC1 and PC2 for replicate samples across all expressed genes, again indicating differential gene expression between IRF4-WT and IRF4-C99R. (**d**) Following Dox-induction over time, bar diagrams show the number of differentially expressed genes that change expression two-fold between IRF4-WT *versus* Mock and IRF4-C99R *versus* Mock. Note, that IRF4-C99R regulates less genes up or down compared to IRF4-WT. (**e**) Four-way Venn diagram shows gene overlap between differentially expressed genes that change expression two-fold after 24 and 48 hours in IRF4-WT *versus* Mock and IRF4-C99R *versus* Mock cells. Left panel, up-regulated genes; right panel, down-regulated genes. Note, that for both classes of genes there is only marginal overlap between IRF4-WT- and IRF4-C99R-regulated genes (marked in orange), whereas both variants regulate individual gene sets (marked in blue). (**f**) Hierarchical clustering of standardized expression levels (z-score) of plasma cell genes. Red colours indicate highly expressed genes. Note, that IRF4-C99R is unable to induce plasma-cell genes, which are induced by IRF4-WT. (**g**) IRF4-WT and IRF4-C99R rescue HRS cells from *IRF4* shRNA-induced cell death. Following shRNA-mediated IRF4 knock-down, L428 HRS cells were transduced with empty vector (control), or cDNA encoding IRF4-WT, IRF4-C99R, or IRF4-C99RS104T. Cell viability is shown normalized to control

transduced cells. Note, that IRF4-C99R variants rescue the cells as efficiently as IRF4-WT from *IRF4* shRNA-induced cell death. Curves show mean viabilities (markers) ± standard errors (transparent tunnels) for n = 4 biologically independent replicates. P values in Supplementary figure 2g were determined by one-sample one-tailed t tests. Source data for Supplementary figure parts 2a and 2g are provided in the Source Data file.

Supplementary Figure 3

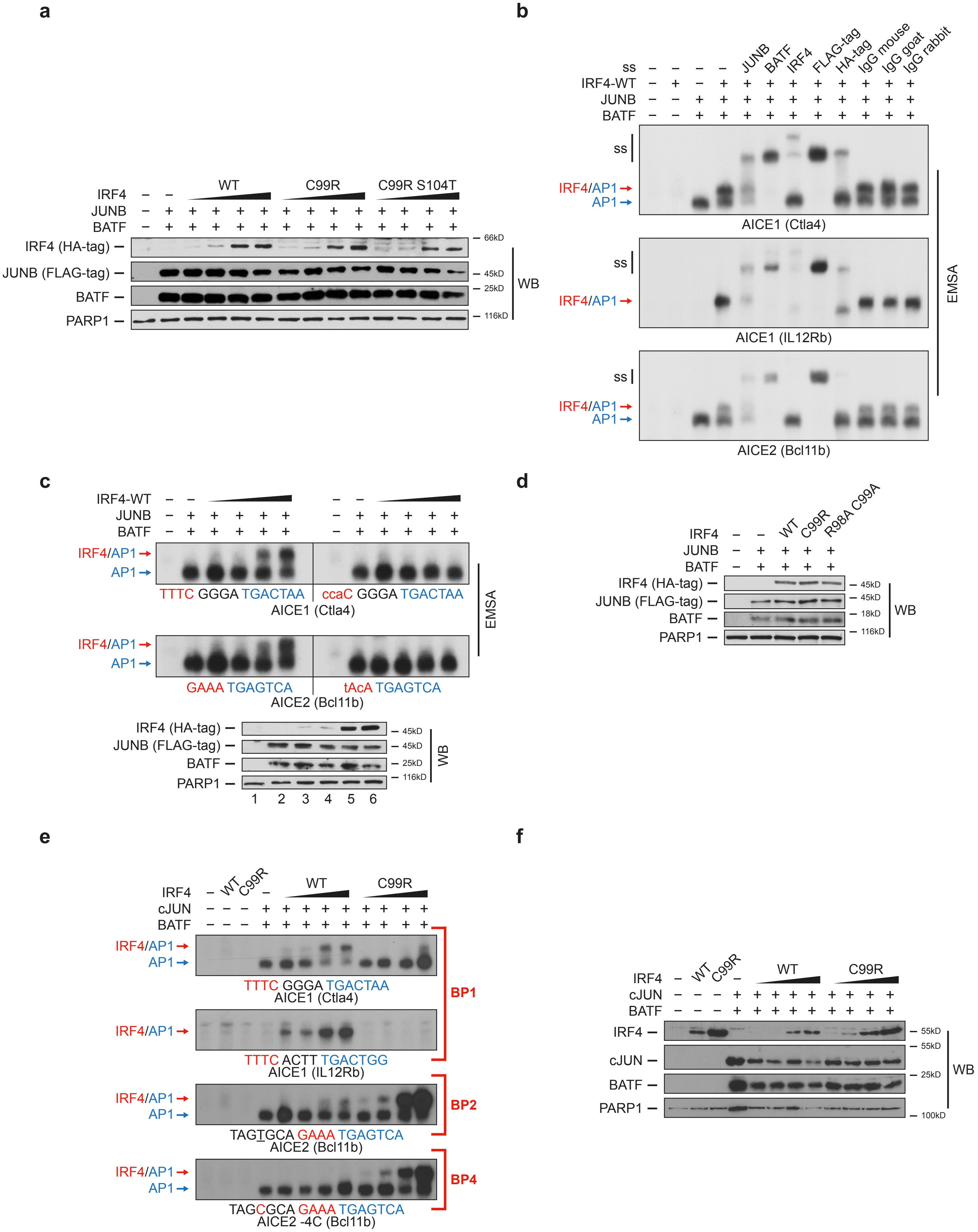

Supplementary Fig. 3. Specificity of JUNB/BATF-IRF4-complexes binding to AICE motifs; EMSA of cJUN/BATF-IRF4 complexes. (**a**) Protein expression controls related to Fig. 1d. Nuclear extracts of HEK293 cells transfected with control plasmid (Mock; '-'), the respective IRF4 variants and JUNB and BATF were analyzed by immunoblotting (WB) for protein expression of IRF4, JUNB, BATF and, as a control, PARP1. Data are representative of at least three independent experiments. (**b**) HEK293 cells were left untreated, or were transfected with IRF4-WT and/or JUNB and BATF, as indicated. Nuclear extracts were analyzed with or without addition of the indicated antibodies for supershift (ss) analyses at AICE1-Ctla4 (top), AICE1-IL12Rb (center), and AICE2-Bcl11b (bottom). Red and blue arrows mark positions of IRF4-JUNB/BATF-DNA and JUNB/BATF-DNA complexes, respectively. One out of three independent experiments is shown. (**c**) HEK293 cells were transfected with control plasmids (Mock, '-'), or were transfected with JUNB and BATF and increasing amounts of IRF4-WT. Nuclear extracts were analyzed by EMSA for binding at AICE1 (Ctla4) and AICE2 (Bcl11b) or variants thereof with mutated half-ISRE site. Note, that mutation of the respective ISRE binding motifs, indicated by small letters within the probe sequence, leads to complete loss of IRF4-JUNB-BATF composite complex formation (upper and lower right panels); binding patterns of JUNB/BATF AP-1 complexes remain unchanged. Protein expression controls are shown underneath. Red and blue arrows mark positions of IRF4-JUNB/BATF-DNA and JUNB/BATF-DNA complexes, respectively. One out of three independent experiments is shown. (**d**) Protein expression controls related to Fig. 1e. Nuclear extracts of HEK293 cells transfected with control plasmid (Mock) or the respective IRF4 variants and JUNB and BATF were analyzed by WB for expression of IRF4, JUNB, BATF and, as a control, PARP1. Data are representative of at least three independent experiments. (**e**) Nuclear extracts of HEK293 cells transfected with cJUN and BATF and increasing amounts of IRF4 variants were analyzed by EMSA for binding to AICE1 (AICE1-Ctla4 and AICE1-IL12Rb), AICE2 (AICE2-Bcl11b) and AICE2^{-4C} probes. Red and blue arrows mark positions

of IRF4-cJUN/BATF-DNA and cJUN/BATF-DNA complexes, respectively. BP, binding pattern. One out of two independent experiments is shown. (**f**) The same nuclear extracts as used in (e) were analyzed by WB for protein expression of IRF4, cJUN, BATF and, as a control, PARP1. One out of two independent experiments is shown. Source data for Supplementary figure 3 are provided in the Source Data file.

Supplementary Figure 4

a

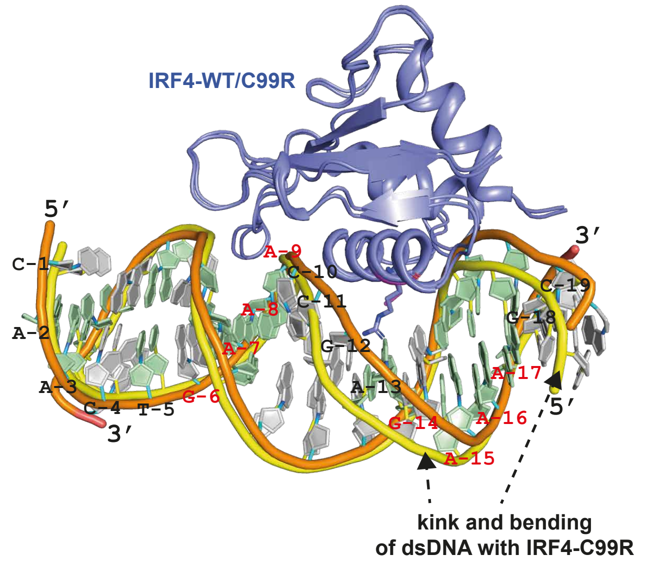

ISRE

5′-[1]C AAC TGA AAC CGA GAA AGC[19]-3′
3′- G TTG ACT TTG GCT CTT TCG -5′

b

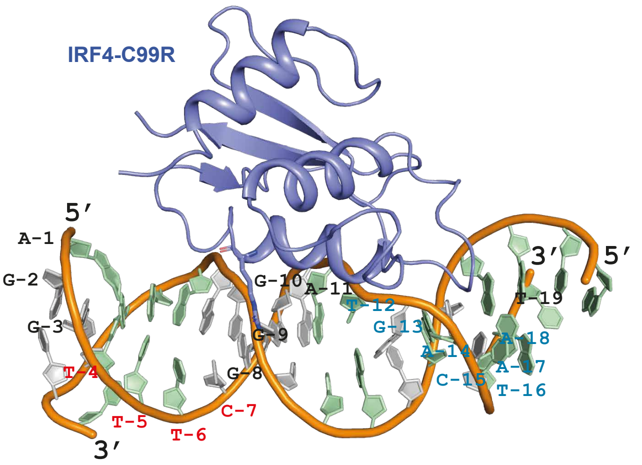

AICE1 (BP1)

5′-[1]AGG TTT CGG GAT GAC TAA T[19]-3′
3′- TCC AAA GCC CTA CTG ATT A -5′

Supplementary Fig. 4. Reference free DNA modelling and docking studies with IRF4-C99R at ISRE and AICE1-DNA motifs. (**a**) Superposition of IRF4-WT (X-ray crystal structure (PDB: 7JM4), orange DNA backbone) and IRF4-C99R (structural model, yellow DNA backbone) with ISRE DNA. IRF4-C99R does not bind to intact ISRE DNA (see Fig. 1g) or needs to push the ISRE DNA for binding to occur (shown here). The unbiased and reference free structural modelling of IRF4-C99R with the ISRE DNA shows bending of the DNA and displacement of the phosphate backbone (displaced DNA strand marked in yellow) to accommodate the recognition helix into the major groove of the DNA. Without this structural re-arrangement of ISRE DNA, binding of IRF4-C99R is difficult. (**b**) Reference free DNA modeling and docking study with IRF4-C99R at AICE1. AICE1-DNA structure modelling shows little available space in the AICE1-DNA major groove resulting in poor interaction of IRF4-C99R with AICE1-DNA. This is notable for the non-canonical mode of the IRF4-C99R interaction with AICE1-DNA, where the major α-helix is docked onto, but not intercalated into the grooves of DNA. Sequences of the ISRE and AICE1 DNA strands in (a) and (b) are shown underneath, with numbered bases of each upper strand shown in the respective image above. Blue/slate, IRF4 (both WT and C99R); orange, DNA phosphate backbone; pale green, dA and dT; grey, dG and dC.

Supplementary Figure 5

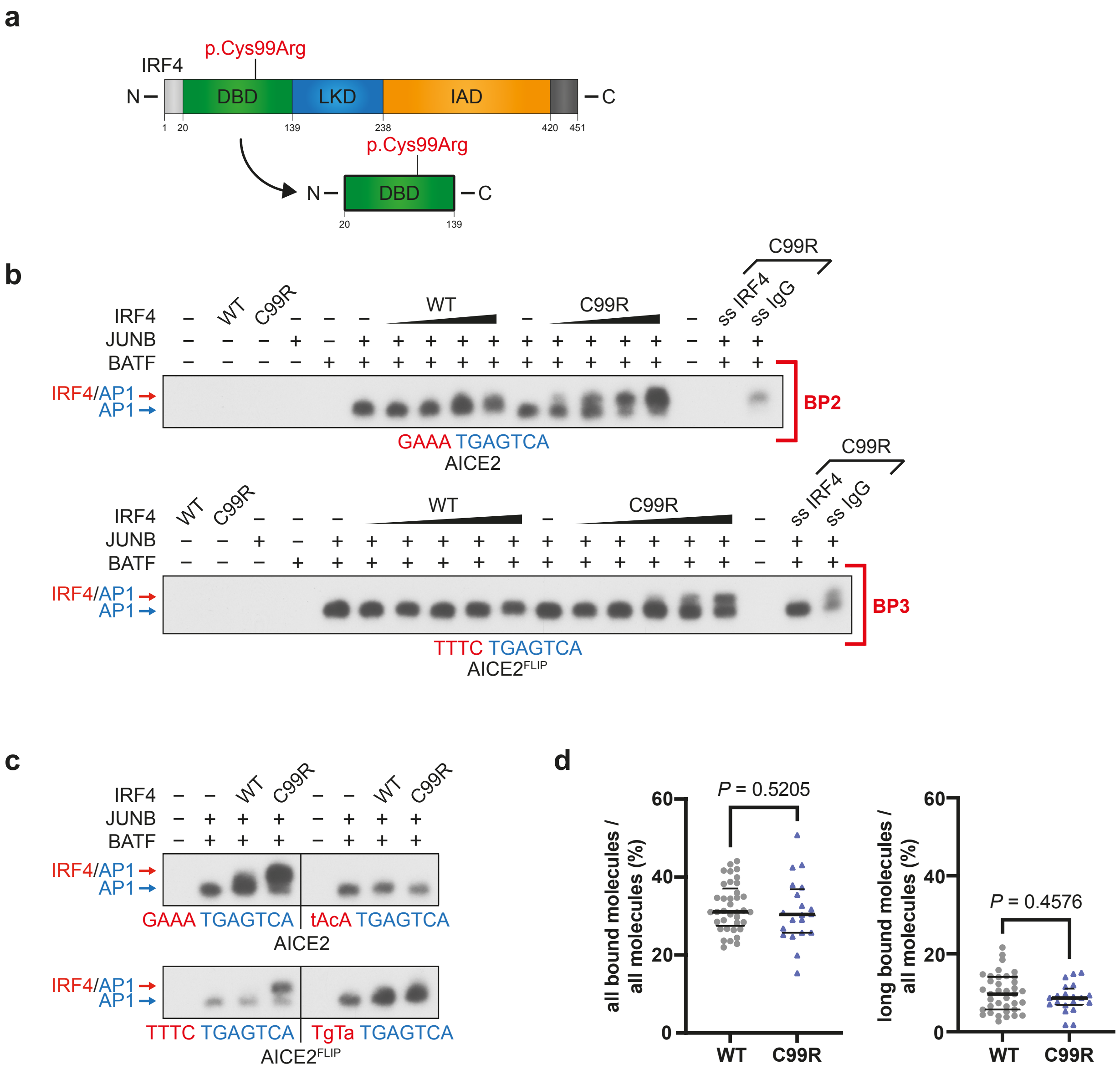

Supplementary Fig. 5. IRF4 DNA binding studies by use of DNA binding domains only and by high-resolution microscopy. (**a**) Schematic of full-length IRF4 (top) and IRF4-DBD (bottom), the latter used in Supplementary Figs. 5b and 5c. (**b**) Recombinant proteins comprising the DNA-binding domains (DBD) of JUNB (AA 269-329) or BATF (AA 28-87) were tested alone or together, or together in combination with increasing amounts of IRF4-WT or IRF4-C99R DBD (AA 20-139), as indicated, for their ability to bind to AICE2 (Bcl11b) (upper panel, BP2) or AICE2^{FLIP} (lower panel, BP3). Red and blue arrows mark positions of IRF4-JUNB/BATF-DNA and JUNB/BATF-DNA complexes, respectively. Far right, supershift analyses by addition of antibody to IRF4 or, as a control, IgG. Note, that IRF4-C99R DBD binds, compared to IRF4-WT, much stronger to AICE2 (Bcl11b), and exclusively to AICE2^{FLIP}. One out of three independent experiments is shown. (**c**) Recombinant proteins, as described in (b) were analyzed for binding to AICE2 (Bcl11b)-WT (upper left) and AICE2 (Bcl11b)-IRFMut (upper right), as well as to AICE2^{FLIP} (lower left) and AICE2^{FLIP}-IRFMut (lower right). Note, that mutation of the IRF motif in each of the probes abolishes formation of IRF4-JUNB/BATF-DNA composite complexes. Positions of the complexes are indicated as described in (b). One out of three independent experiments is shown. (**d**) Determination of IRF4-WT and -C99R binding fractions. Fractions of all bound SiR-HaloTag-IRF4-WT and IRF4–C99R molecules (left) and molecules bound for > 2s (right) as determined by single-molecule fluorescence microscopy with interlaced time-lapse illumination. *P* values in Supplementary figure 5d were determined by unpaired, non-parametric *t* test (Mann-Withney-test). Source data for Supplementary figure parts 5b-d are provided in the Source Data file.

Supplementary Figure 6

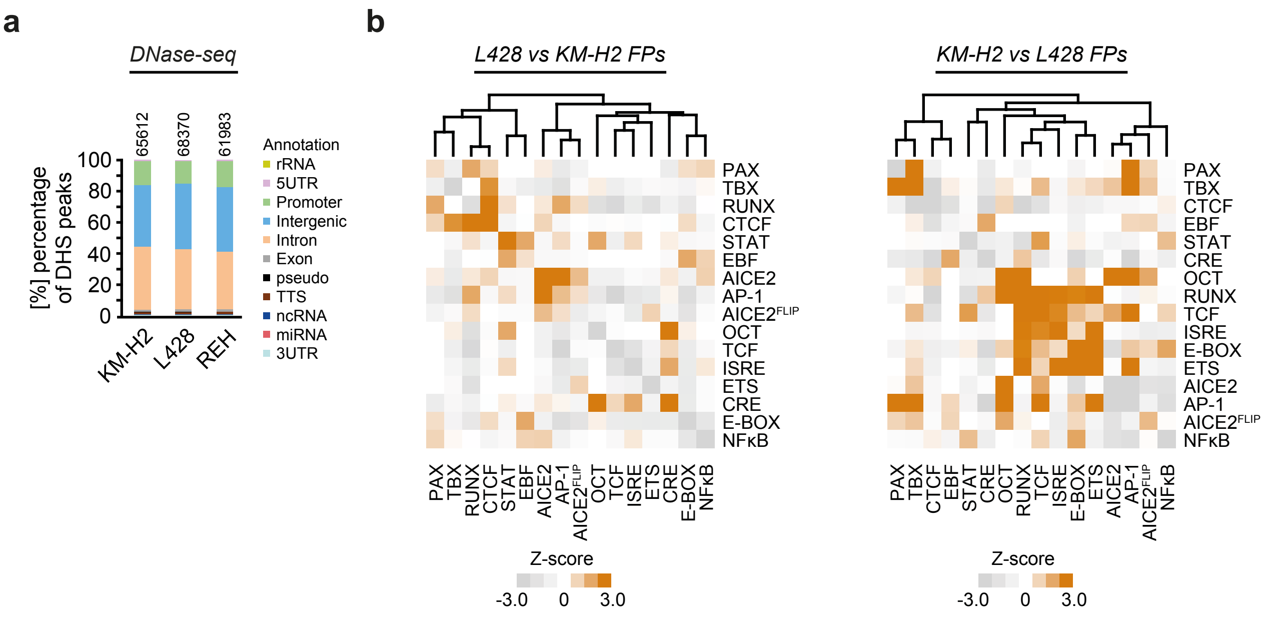

Supplementary Fig. 6. Genome-wide localization of DNaseI-Seq peaks and co-occurring clustering of AICE2. (**a**) Genome-wide localization of DNase-seq peaks in KM-H2$^{\mathrm{IRF4\text{-}WT}}$, L428$^{\mathrm{IRF4\text{-}C99R}}$ and REH cells. Note, that most peaks are intergenic or intronic, with about 20% of DHSs showing promoter enrichment. (**b**) Heatmap detailing z-scores of self- and co-occurrence enrichments in L428 *versus* KM-H2 (left) or KM-H2 *versus* L428 (right) footprints for 16 motifs corresponding to TFs important to B cell and HL gene regulation. |Z-scores| above 1.96 represent $\geq 2\sigma$ enrichment. All DGF/DNase-Seq values are from two averaged replicates per condition.

Supplementary Figure 7

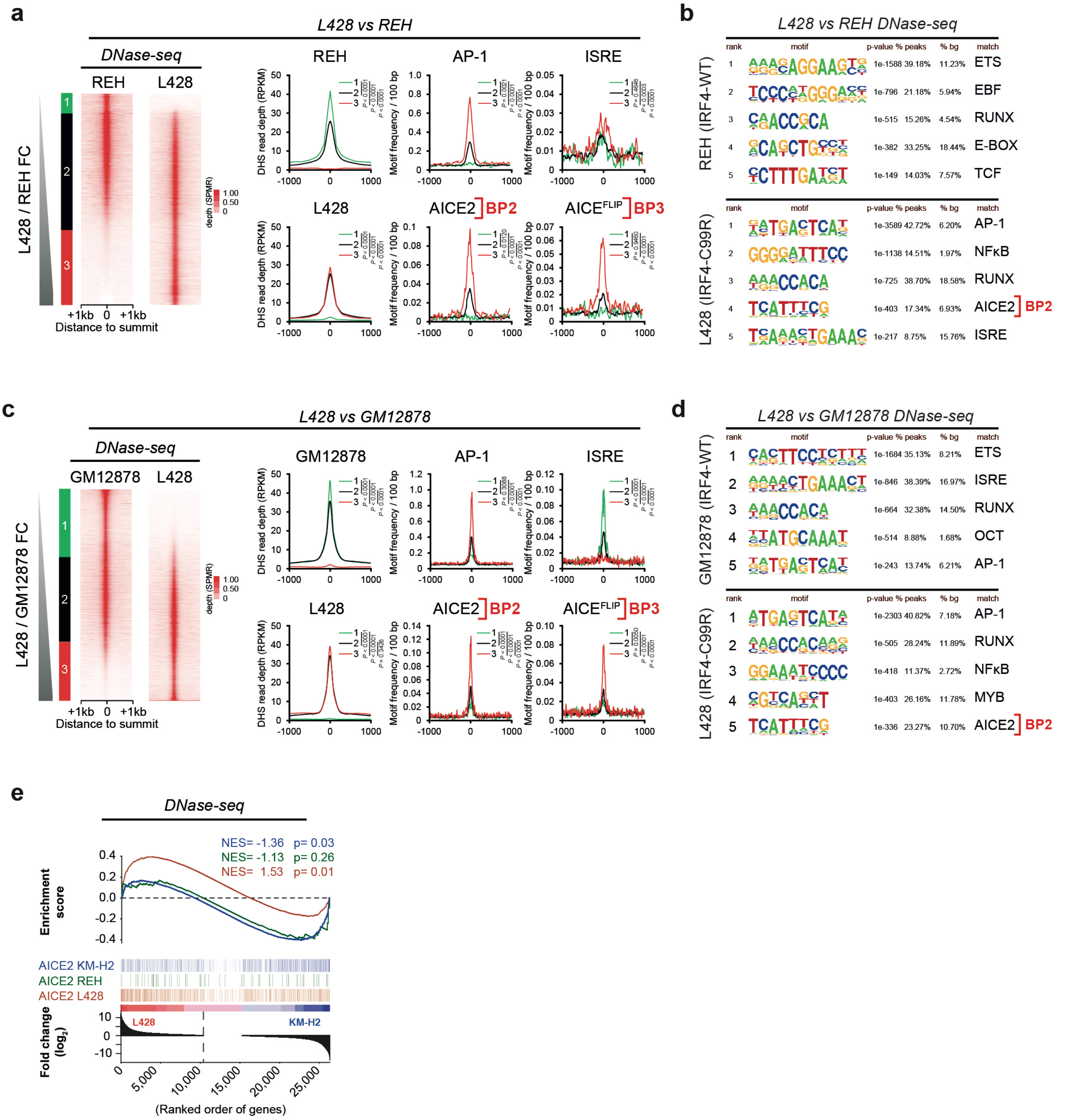

Supplementary Fig. 7. AICE2 motifs are enriched in IRF4-C99R HL cells and are associated with increased gene expression. (**a**) Heatmaps showing the DNase-seq fold change analysis (left) between L428$^{IRF4\text{-}C99R}$ HL and non-Hodgkin REH cells and corresponding DNase-Seq motif average profiles (right). (**b**) HOMER *de novo* motif discovery-results from classes 1 (REH-specific, top) and 3 (L428$^{IRF4\text{-}C99R}$-specific, bottom), defined in (a). (**c**, **d**) Same as (a, b) but comparing L428 with GM12878 DNase-Seq. Note, that both comparisons reveal AICE2 and AICE2^{FLIP} motifs as specifically enriched in L428 $^{IRF4\text{-}C99R}$ cells. (**e**) GSEA of footprinted L428$^{IRF4\text{-}C99R}$, KM-H2$^{IRF4\text{-}WT}$, and REH AICE2 motif presence *vs* L428 / KM-H2 RNA-seq fold change. Note, that footprinted AICE2 motif presence is associated with increased gene expression for L428 followed by KM-H2, but not in REH cells. All DNase-Seq/RNA-Seq values are from two average replicates per condition. *P* values in Supplementary figures 7a and 7c were determined by two-tailed unpaired *t* test without adjustment for multiple comparisons.

Supplementary Figure 8

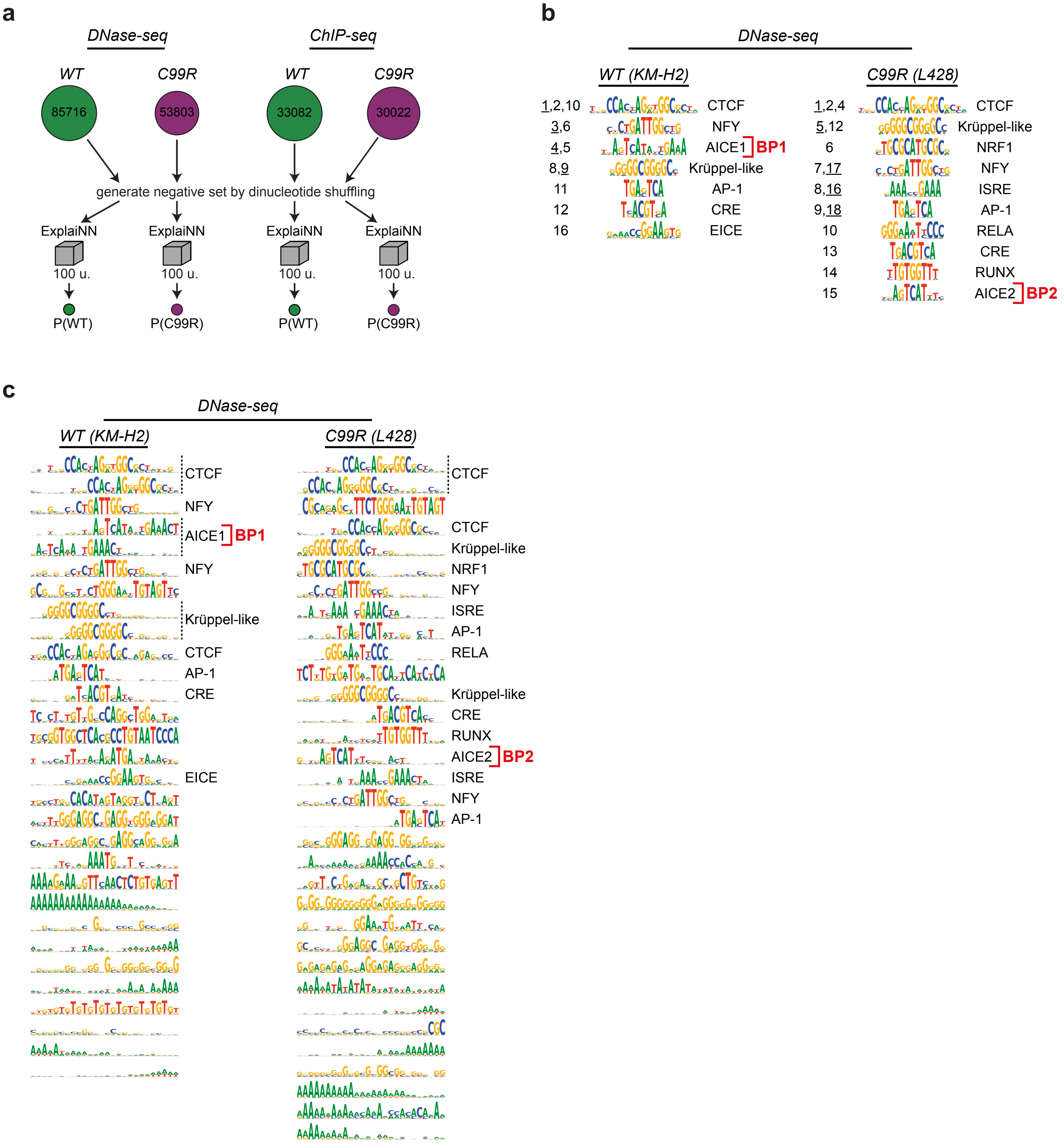

Supplementary Fig. 8. (**a**) The four ExplaiNN models trained in this work. (**b**) *De novo* motif discovery in specific DHSs datasets of KM-H2^{IRF-WT} and L428$^{IRF4-C99R}$ using ExplaiNN. Motifs are ranked by their importance (left). When more than one motif of the same class was identified, the rank of the displayed motif is underlined. Only motifs that could by annotated with a biological representation are shown. (**c**) List of all motifs identified in the DHS data sets by ExplaiNN in (b) ranked by their importance. All DNase-Seq values are from two averaged replicates per condition.

Supplementary Figure 9

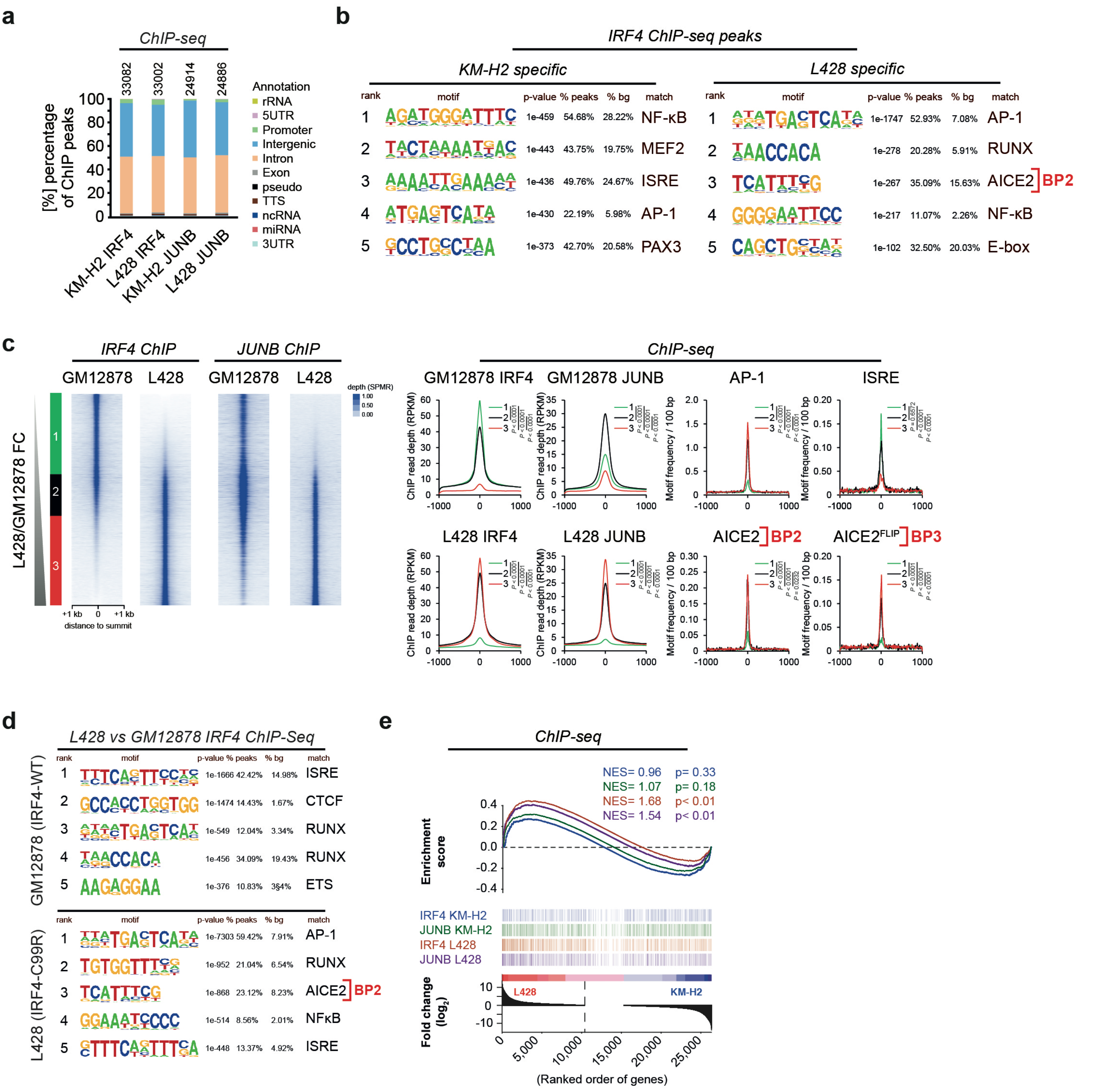

Supplementary Fig. 9. AICE2 motifs are associated with increased IRF4/JUNB co-localization in IRF4-C99R HL cells. (**a**) Genome-wide localization of IRF4, JUNB ChIP-Seq peaks in KM-H2 and L428 cells. Most peaks are intergenic and intronic. (**b**) HOMER motif discovery results in KM-H2 (left)- and L428 (right)-specific IRF4 ChIP peaks defined in Fig. 2g. (**c**) Heatmaps showing IRF4 ChIP fold change (FC) analysis (blue, leftmost) between L428 and GM12878 cells and corresponding JUNB ChIP-Seq signal (blue, rightmost), as well as corresponding ChIP-seq motif average profiles (right). Note, that AP-1, AICE2 (BP2) and $AICE^{FLIP}$ (BP3) signals are highest in $L428^{IRF4\text{-}C99R}$-specific peaks. (**d**) HOMER *de novo* motif discovery-results from classes 1 and 3 (top and bottom, respectively) in GM12878 (top) and L428 (bottom)-specific IRF4 ChIP-seq peaks defined in (c). (**e**) GSEA of the top 1,000 differential IRF4 and JUNB L428 and KM-H2 ChIP-seq peaks (left and right, respectively) against corresponding L428 / KM-H2 RNA-seq fold change (FC). Note, that IRF4 and JUNB ChIP peaks are only significantly associated with increased gene expression in $L428^{IRF4\text{-}C99R}$ cells. All ChIP-Seq/RNA-Seq values are from two averaged replicates per condition. *P* values in Supplementary figure 9c (right) were determined by two-tailed unpaired *t* test without adjustment for multiple comparisons.

Supplementary Figure 10

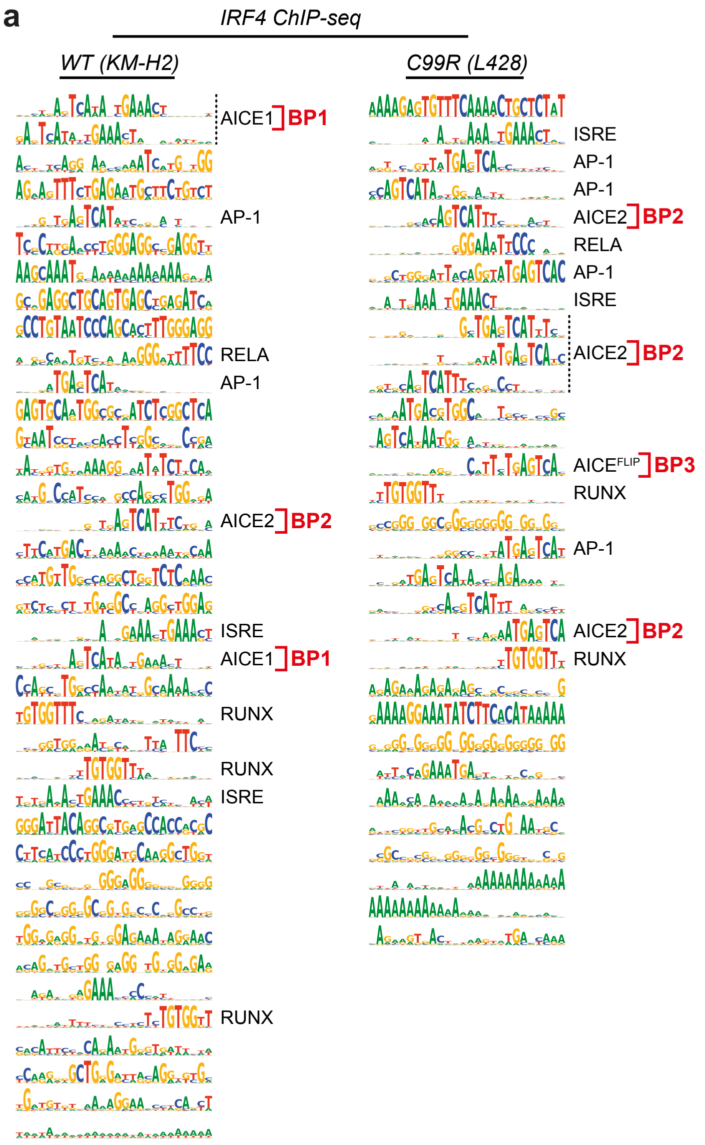

Supplementary Fig. 10. Increased AICE2 motif frequencies in IRF4-C99R HL cells from neural-network based motif discovery approaches. (**a**) Full report of motif discovery results of the ChIP-seq data sets using ExplaiNN, detailing sub-motifs making aggregate motifs described in Fig. 2i. Note, that AICE1 (BP1) is only identified in KM-H2$^{IRF4\text{-}WT}$ cells, whereas AICE2 BP2 is overrepresented and AICE2 BP3 exclusively found in L428$^{IRF\text{-}C99R}$ cells. All ChIP-Seq values are from two averaged replicates per condition.

Supplementary Figure 11

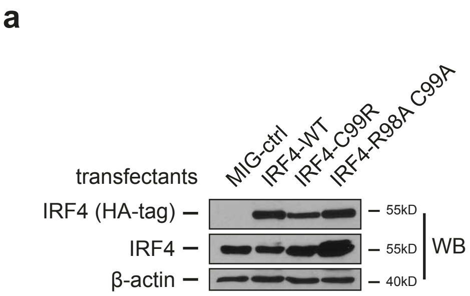

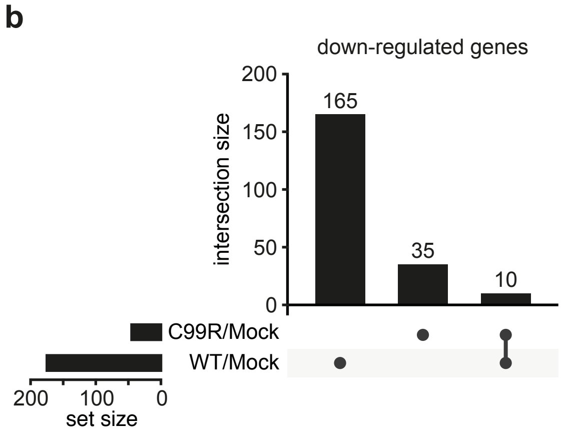

Supplementary Fig. 11. Expression controls of transduced C57BL/6 mouse splenic B cells and IRF4-C99R down-regulated genes. (**a**) C57BL/6 mouse splenic B cells transduced with MIG control retrovirus (MIG-ctrl; Mock), IRF4-WT, IRF4-C99R or, as a further control, IRF4-R98AC99A, were analyzed by immunoblotting for expression levels of IRF4 by use of HA-tag and IRF4 antibody, respectively. β-actin was analyzed as a control. Data are representative of three independent experiments. (**b**) IRF4-C99R and IRF-WT regulated genes show only low overlap, as shown in UpSet plots for down-regulated genes. Source data for Supplementary figure part 11a are provided in the Source Data file.

Supplementary Figure 12

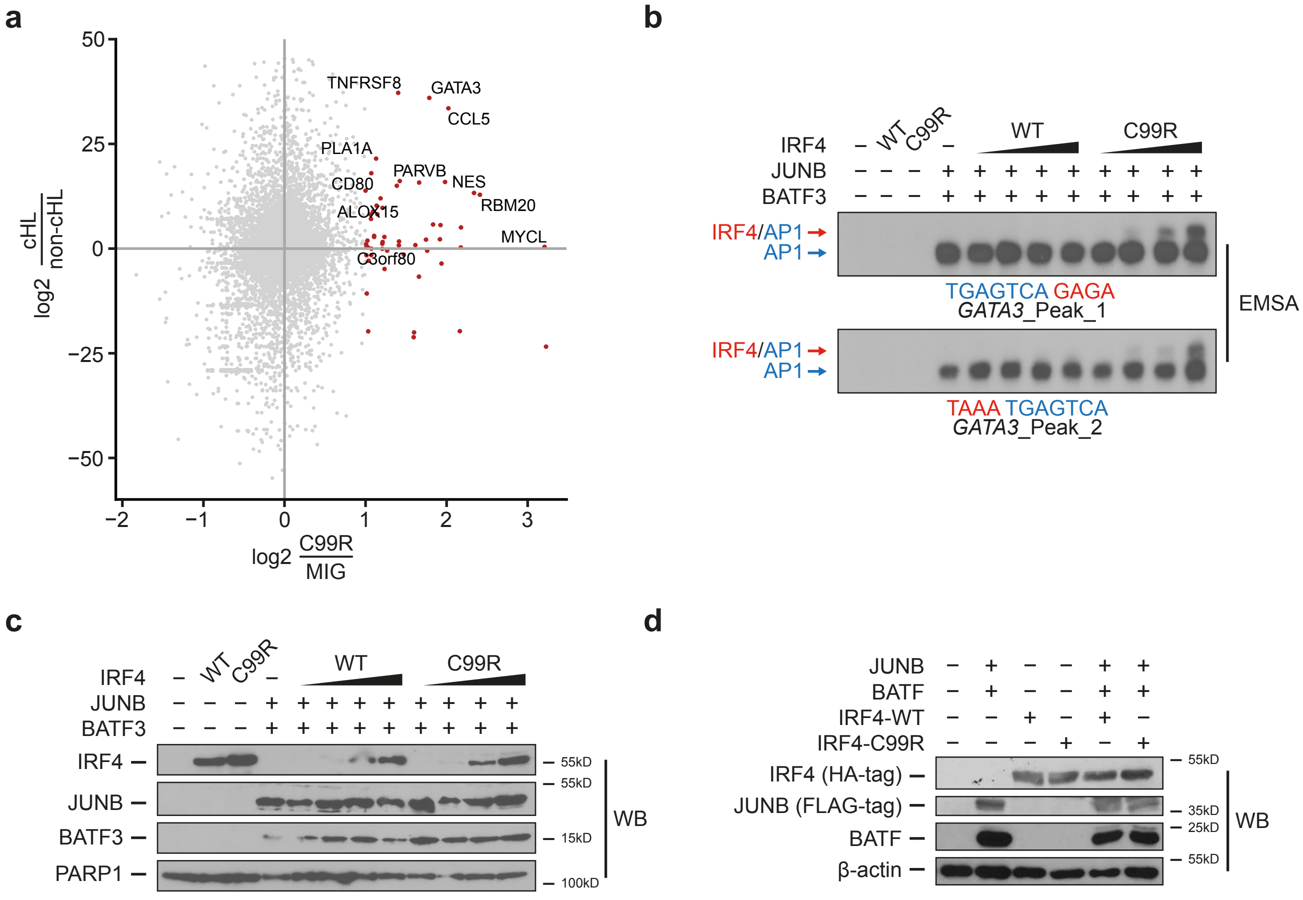

Supplementary Fig. 12. IRF4-C99R up-regulated genes encompass cHL hallmark genes; EMSA of JUNB/BATF3-IRF4 complexes; expression controls for reporter gene studies. (**a**) Comparison of fold changes between IRF4-C99R-induced genes in mouse splenic B cells with differentially expressed genes of Hodgkin and non-Hodgkin cell lines based on RNA-seq analyses. The spearman correlation between the log2 fold changes of cHL (L428, L1236, KM-H2, U-HO1, L540Cy) / non-cHL (REH, NAMALWA, SU-DHL-4, BJAB) *versus* C99R / MIG was 0.1633, with a *P* value of less than 10^-5. The number of genes compared was 17193. (**b**) HEK293 cells were control transfected (-), or transfected with IRF4-WT, IRF4-C99R, JUNB and BATF3, or combinations thereof, as indicated. Nuclear extracts were analyzed for DNA-binding activity at *GATA3*Peak_1 and *GATA3*Peak_2 sites, as indicated. Note, that IRF4/JUNB/BATF3_complexes are only detected at the sites in the presence of IRF4-C99R, whereas IRF4-WT does not bind at these sites. EMSA data show one out of two independent experiments. (**c**) The same nuclear extracts as used in (b) were analyzed by immunoblotting (WB) for protein expression of IRF4, JUNB, BATF3 and, as a control, PARP1. One out of two independent experiments is shown. (**d**) Protein expression controls related to Fig. 4d. Whole cell extracts of HEK293 cells transfected with control plasmid (Mock, '-'), JUNB, BATF, IRF4-WT or IRF4-C99R alone or in combination, as indicated, were analyzed by immunoblotting for expression of JUNB, BATF, IRF4 and, as a control, β-actin. Data are representative of three independent experiments. Source data for Supplementary figure parts 12b-d are provided in the Source Data file.

Supplementary Table 1. Features of the IRF4 mutation.

Variant Annotation	
**Chromosome**	6
**Genomic Position (GRCh38)**	394899
**cDNA Position (NM_001195286)**	295
**Nucleotide Reference**	T
**Nucleotide variant**	C
**Protein Variant**	Cys99Arg; C99R
**dbSNP153**	No entry
**gnomAD**	No entry
**COSMIC (v96)**	Somatic report, 9 entries (Mutation ID COSV66704715)

In silico Pathogenicity Prediction Models	
**CADD**	24.0
**SIFT**	Deleterious (0)
**Polyphen-2**	Probably damaging (1)
**LRT**	Deleterious (0)
**MutationTaster**	Disease causing (1)
**PROVEAN**	Deleterious (-11.56)
**MetaSVM**	Deleterious (1.011)
**M-Cap**	Possibly pathogenic (0.87)
**fathmm_MKL-coding**	Deleterious (0.942)

Supplementary Table 2. *IRF4* exon 3 mutation analysis of HRS cells.

sample no	disease stage	subtype	EBV	mutated sequences per total sequences*	c.295T>C
**1**	primary	NS	-	0 / 4	wt
**2**	primary	NS	-	0 / 2	wt
**3**	primary	MC	-	0 / 2	wt
**4**	primary	NS	-	0 / 2	wt
**5**	primary	NS	-	0 / 2	wt
**6**	primary	MC	+	0 / 2	wt
**7**	primary	NS	-	0 / 4	wt
**8**	primary	NS	-	0 / 2	wt
**9**	primary	NS	-	0 / 3	wt
**10**	primary	NS	-	0 / 2	wt
**11a**	primary	NS	-	2 / 4	mut
**11b**	relapse	NS	-	2 / 3	mut**
**12**	relapse	NS	-	0 / 4	wt
**13**	relapse	NS	-	0 / 2	wt
**14**	relapse	NS	-	0 / 3	wt
**15**	relapse	NS	-	0 / 4	wt
**16**	relapse	NS	-	2 / 3	mut
**17**	relapse	MC	+	0 / 3	wt
**18**	relapse	NS	-	2 / 5	mut
**19**	relapse	LR***	+	0 / 3	wt
**20**	relapse	MC	+	0 / 2	wt

NS, nodular sclerosis; MC, mixed cellularity; LR, lymphocyte-rich; wt, wild type; mut, mutated.

* 2-4 informative sequences from pools of 10 microdissected HRS cells were analyzed per case.

**PCR products were cloned into pGEMTeasy and sequenced from plasmid DNA to identify 4/27 mutated sequences.

***subtype at primary diagnosis

Supplementary Table 3. Validation report for the predicted structure of C99R-IRF4 with ISRE and AICE1 DNA fragments. Up to 100 models were generated for each of the complexes and these models were classified into 6 to 12 clusters of varying sizes. The cluster size for the lowest energy cluster is shown in the table. The RMSD refers to the entire complex. The tabulated scores are primarily derived from docking scores (HADDOCK) while additional scores denote RMSD, electro-static potential, van der Waals or buried interphase and the z-scores of the predicted complexes. The Z-score indicates the cluster's HADDOCK score from the average of all clusters. A lower Z-score denotes a superior model.

	**ISRE-C99R**	**AICE1-C99R**
**HADDOCK score**	-163.2 +/- 9.7	-95.6 +/- 0.2
**Cluster size**	28	12
**RMSD**	1.0 +/- 0.6	18.2 +/- 0.0
**Van der Waals**	-86.5 +/- 8.0	-46.5 +/- 1.1
**Electrostatic Energy**	-528.0 +/- 38.7	-293.2 +/- 7.4
**Desolvation Energy**	25.0 +/- 2.8	8.0 +/- 0.7
**Restraints violation**	38.8 +/- 24.3	15.7 +/- 16.4
**Buried Surface Area**	2315.8 +/- 170.7	1094.0 +/- 19.5
**Z-Score**	-1.8	+0.2

Supplementary Table 4. Oligonucleotides used in the present study.

Probes for EMSA		
ISRE (ISG15)	forward	AGCTGGGAAAGGGAAACCGAAACTG
	reverse	AGCTCAGTTTCGGTTTCCCTTTCCC
AICE1 (Ctla 4)	forward	AGCTCTTGCCTTAGAGGTTTCGGGATGACTAATACT GTA
	reverse	AGCTTACAGTATTAGTCATCCCGAAACCTCTAAGGC AAG
AICE1 (Il12Rb)	forward	AGCTGCTTTTGCTTTCACTTTGACTGGCCTGGAGAC AATGAGTT
	reverse	AGCTAACTCATTGTCTCCAGGCCAGTCAAAGTGAAA GCAAAAGC
AICE2 (Bcl11b)	forward	AGCTTAGTGCAGAAATGAGTCAGAGATCAAAGAAG
	reverse	AGCTCTTCTTTGATCTCTGACTCATTTCTGCACTA
SP1	forward	AGCTATTCGATCGGGGCGGGGCGAGC
	reverse	AGCTGCTCGCCCCGCCCCGATCGAAT
AICE2 5´TTTC	forward	AGCTTAGTGCATTTCTGAGTCAGAGATCAAAGAAG
	reverse	AGCTCTTCTTTGATCTCTGACTCAGAAATGCACTA
AICE2 3´GAAA	forward	AGCTTAGTGCATGAGTCAGAAAGAGATCAAAGAAG
	reverse	AGCTCTTCTTTGATCTCTTTCTGACTCATGCACTA
AICE2 3´TTTC	forward	AGCTTAGTGCATGAGTCATTTCGAGATCAAAGAAG
	reverse	AGCTCTTCTTTGATCTCGAAATGACTCATGCACTA
AICE2-4C (Bcl11b)	forward	AGCTTAGCGCAGAAATGAGTCAGAGATCAAAGAAG
	reverse	AGCTCTTCTTTGATCTCTGACTCATTTCTGCGCTA
AICE2 (Bcl11b_short)	forward	AGTGCAtAcATGAGTCAGAGATC
	reverse	AGGATCTCTGACTCATgTaTGCA
AICE2 (Bcl11b_short_IRFmut)	forward	AGTGCAGccATGAGTCAGAGATC
	reverse	CGGATCTCTGACTCATggCTGCA
AICE^{FLIP} (Bcl11b_5´TTTC_FLIP_short)	forward	AGTGCATTTCTGAGTCAGAGATC
	reverse	AGGATCTCTGACTCAGAAATGCA
AICE^{FLIP} (Bcl11b_5´TTTC_FLIP_short_IRFmut)	forward	AGTGCATgTaTGAGTCAGAGATC
	reverse	AGGATCTCTGACTCAtAcATGCA

GATA3Peak_1	forward	AGCTACTGAATGAGTCAGAGAGGCCCAAA
	reverse	AGCTTTTGGGCCTCTCTGACTCATTCAGT
GATA3Peak_2	forward	AGCTAGTCATAAATGAGTCATGGC
	reverse	AGCTGCCATGACTCATTTATGACT
GATA3Peak_3	forward	AGCTAATGCAGGAATGACTCACTTG
	reverse	AGCTCAAGTGAGTCATTCCTGCATT
Primers for Site-Directed Mutagenesis		
IRF4mut_C99R	forward	GAAGACGCGCCTGCGGCGCGCTTTGAACAAGAGCAATG
	reverse	CATTGCTCTTGTTCAAAGCGCGCCGCAGGCGCGTCTTC
IRF4mut_C99RS104T	forward	CGCCTGCGGCGCGCTTTGAACAAGACCAATGACTTTG
	reverse	CAAAGTCATTGGTCTTGTTCAAAGCGCGCCGCAGGCG
IRF4mut_C99A	forward	GGAAGACGCGCCTGCGGGCCGCTTTGAACAAGAG
	reverse	CTCTTGTTCAAAGCGGCCCGCAGGCGCGTCTTCC
IRF4mut_R98A	forward	GAAGACGCGCCTGGCGTGCGCTTTGAACAAGAG
	reverse	CTCTTGTTCAAAGCGCACGCCAGGCGCGTCTTC
IRF4mut_R98AC99A	forward	GGAAGACGCGCCTGGCGGCCGCTTTGAACAAGAGCAATG
	reverse	CATTGCTCTTGTTCAAAGCGGCCGCCAGGCGCGTCTTCC

Supplementary Table 5. Antibodies used in the present study.

**ANTIBODIES**	**COMPANY**	**IDENTIFIER**
Antibodies for EMSA (all used 1 – 3 μl/reaction)		
IRF4 (M-17)	Santa Cruz Biotechnology	sc-6059
JUNB (N-17)	Santa Cruz Biotechnology	sc-46
BATF (clone WW8)	Santa Cruz Biotechnology	sc-100974
HA-tag (clone C29F4)	Cell Signaling Technology	#3724
Anti-Flag (clone M2)	Sigma-Aldrich	F1804
BATF (clone D7C5)	Cell Signaling Technology	#8638
IgG1 mouse (clone #11711)	R&D Systems	MAB002
IgG rabbit	R&D Systems	AB-105-c
IgG goat	R&D Systems	AB-108-c
Antibodies for Western Blot		
IRF4 (M-17; dilution 1:1000)	Santa Cruz Biotechnology	sc-6059
IRF4 (clone D9P5H; dilution 1:1000)	Cell Signaling Technology	#15106
HA-probe (Y-11; dilution 1:1000)	Santa Cruz Biotechnology	sc-805
HA-tag (clone C29F4; dilution 1:1000)	Cell Signaling Technology	#3724
Anti-Flag (clone M2; dilution 1:1000)	Sigma-Aldrich	F1804
c-JUN (H-79; dilution 1:1000)	Santa Cruz Biotechnology	sc-1694
BATF (clone D7C5; dilution 1:500)	Cell Signaling Technology	#8638
BATF (clone WW8; dilution 1:500)	Santa Cruz Biotechnology	sc-100974
SNFT/BATF3 (M-13; dilution 1:500)	Santa Cruz Biotechnology	sc-162246x
PARP1 (clone F-2; dilution 1:1000)	Santa Cruz Biotechnology	sc-8007
β-actin (clone AC-74; dilution 1:1000)	Sigma-Aldrich	#A5316

Antibodies for FACS		
CD138-PE (dilution 1:400)	Biolegend	142504
PerCP/cy5.5 Anti-mouse B220 (dilution 1:400)	Biolegend	103235
