# Supplementary material for: Transcriptional reprogramming by mutated IRF4 in lymphoma
Source: Nat Commun. 2023 Nov 7;14:6947. doi: 10.1038/s41467-023-41954-8 (PMC10630337; doi:10.1038/s41467-023-41954-8)
Supplement: Supplementary file 3 — Reporting Summary [file 41467_2023_41954_MOESM3_ESM.pdf]

nature portfolio

Corresponding author(s): Prof. Dr. Stephan Mathas
Last updated by author(s): Aug 23, 2023

Reporting Summary

Nature Portfolio wishes to improve the reproducibility of the work that we publish. This form provides structure for consistency and transparency in reporting. For further information on Nature Portfolio policies, see our Editorial Policies and the Editorial Policy Checklist.

Statistics

For all statistical analyses, confirm that the following items are present in the figure legend, table legend, main text, or Methods section.

n/a	Confirmed	
☐	☒	The exact sample size (*n*) for each experimental group/condition, given as a discrete number and unit of measurement
☐	☒	A statement on whether measurements were taken from distinct samples or whether the same sample was measured repeatedly
☐	☒	The statistical test(s) used AND whether they are one- or two-sided<br>*Only common tests should be described solely by name; describe more complex techniques in the Methods section.*
☒	☐	A description of all covariates tested
☐	☒	A description of any assumptions or corrections, such as tests of normality and adjustment for multiple comparisons
☐	☒	A full description of the statistical parameters including central tendency (e.g. means) or other basic estimates (e.g. regression coefficient) AND variation (e.g. standard deviation) or associated estimates of uncertainty (e.g. confidence intervals)
☐	☒	For null hypothesis testing, the test statistic (e.g. *F*, *t*, *r*) with confidence intervals, effect sizes, degrees of freedom and *P* value noted<br>*Give P values as exact values whenever suitable.*
☒	☐	For Bayesian analysis, information on the choice of priors and Markov chain Monte Carlo settings
☒	☐	For hierarchical and complex designs, identification of the appropriate level for tests and full reporting of outcomes
☐	☒	Estimates of effect sizes (e.g. Cohen's *d*, Pearson's *r*), indicating how they were calculated

Our web collection on statistics for biologists contains articles on many of the points above.

Software and code

Policy information about availability of computer code

Data collection

*DNAse-seq: Libraries were sequenced at 400 million reads per library using an Illumina HiSeq 2000 system.
*ChIP-seq: Libraries were sequenced at 50 million reads per library using an Illumina HiSeq 2000 system.
*Single-molecule fluorescence microscopy: Localization of fluorescent molecules within an image and tracking of molecules accross consecutive images was performed by use of TrackIt v1.0.1.
*RNA-seq of human lymphoma cell lines: Libraries were sequences on the Illumina Hiseq2500.
*RNA-seq of mouse splenic B cells: Libraries were sequenced by use of Illumina HighSeq 4000.
*BJAB microarray gene expression analysis was performed by use of Human HAT-12_v4 Bead Chips (Affymetrix).

Data analysis

*STAR v2.3.0
*featureCounts v2.0.0
*DESeq2 v1.14.1
*GSEA v3.0
*HiSeq Analysis Software 2.0 (Illumina)
*bcl2fastq v2.16.0 (Illumina)
*bowtie2 v2.1.0
*samtools sort v1.1
*macs2 v2.1.0
*bedtools intersect v2.19.0
*annotatePeaks v4.6
*Java TreeView v1.1.4
*ChIPpeakAnno make VennDiagram v.1.12.0

*gplots heatmap.2 v2.17.0
*homer v4.6
*pyDNase wellington_footprints v0.2.6
*pyBedtools 0.9.0
*SALMON v0.9.1
*ComplexHeatmap v2.10.0
*DESeq2 v1.34.0
*tximport v1.22.0
*UpSetR v1.4.0
*BEDTools slop v2.30.0
*BiasAway v3.3.0
*scikit-learn v0.24.2
*SciPy v1.7.1
*Primer3 v4.1.0
*SeqScape v2.5
*Trackit v1.0.1
*Graphpad prism v9.0.1
*HADDOCK 2.2
*PyMOL v2.5
*FlowJo v9.9.6
*R v4.0.3
*R v4.1
*Prism v9.0.2
*ExplaiNN: ExplaiNN is open-source software distributed under the MIT license. It is available on GitHub (https://github.com/wassermanlab/ExplaiNN) accompanied by a collection of Python scripts that were used to train and interpret the models. BEDTools (version 2.30.0) was used for processing BED files. BiasAway (version 3.3.0) was used to generate negative datasets. ExplaiNN models were implemented in PyTorch (version 1.9.0). Scikit-learn (version 0.24.2) was used for data processing and statistical analyses). Tomtom (version 5.3.0) was used to map convolutional filters to JASPAR profiles.

For manuscripts utilizing custom algorithms or software that are central to the research but not yet described in published literature, software must be made available to editors and reviewers. We strongly encourage code deposition in a community repository (e.g. GitHub). See the Nature Portfolio guidelines for submitting code & software for further information.

Data

Policy information about availability of data

All manuscripts must include a data availability statement. This statement should provide the following information, where applicable:

- Accession codes, unique identifiers, or web links for publicly available datasets
- A description of any restrictions on data availability
- For clinical datasets or third party data, please ensure that the statement adheres to our policy

Data sets generated in the study are deposited in public databases. Gene Expresssion Omnibus (GEO) superseries accession GSE211445 contains: DNase-Seq data: GSE211441; ChIP-Seq data: GSE211443; HL and NHL cell line RNA-Seq data: GSE211444; BJAB cells with TET-inducible control, IRF4-WT and IRF4-C99R Illumina BeadChip HT-12 V4.0 expression arrays: GSE211913.
RNA-Seq data of mouse splenic B cells are deposited in ArrayExpress database under ID E-MTAB-12522.
Reads for IRF4 and JUNB GM12878 ENCODE ChIP-Seq datasets and GM12878 ENCODE DNase-Seq were retrieved from the Sequence Read Archive (SRA).
High-throughput sequencing data of the PMBCL cohort will be available upon request from F. Damm (Berlin).
Original blots of the study are provided in the Source Data file of the study.

Research involving human participants, their data, or biological material

Policy information about studies with human participants or human data. See also policy information about sex, gender (identity/presentation), and sexual orientation and race, ethnicity and racism.

Reporting on sex and gender	Classic Hodgkin lymphoma, the main lymphoma entity for which we show data in our manuscript, shows only minor differences in sex distributions (ratio male : female – 1,3 : 1), with an even equal distribution between men and woman in young adulthood. Thus, a clear-cut sex-based phenotypic bias is unlikely. In our study we thus did not specifically select for sex or performed gender-based analyses.<br><br>Patient material was analyzed retrospectively. Thereby, anonymized primary lymphoma samples were selected on a random base for the immunohistochemistry analyses. Given the large number of cases analysed, it is conceivable that our data reflect the overall distribution of male and female cases within the general population. Selection of materials for lymphoma single cell analyses as well as of the cell lines was primarily determined by the limited availability of respective materials within the community.
Reporting on race, ethnicity, or other socially relevant groupings	Informations on race, ethnicity, or other socially relevant groupings were not collected.
Population characteristics	We used in our study anonymized specimens which were analyzed retrospectively. We did not collect data on population characteristic. Given the large number of cases analyzed, it is conceivable that our data reflect the main distributions in the general population.

Recruitment	Patient material was analyzed retrospectively. Anonymized specimens were provided by the University Cancer Center Frankfurt (UCT; Germany), the Hematopathology Section of Christian-Albrechts-University Kiel (Germany) and the Lymphoma Reference Centre at the Institute of Pathology, University of Würzburg (Germany).
Ethics oversight	The study was approved by the institutional review boards and local Ethics Committees of the Universities Würzburg and Frankfurt/Main as well as Charité - Universitätsmedizin Berlin (SHN-06-2018; 15-6184-BO; EA2/087/16).

Note that full information on the approval of the study protocol must also be provided in the manuscript.

Field-specific reporting

Please select the one below that is the best fit for your research. If you are not sure, read the appropriate sections before making your selection.

☒ Life sciences ☐ Behavioural & social sciences ☐ Ecological, evolutionary & environmental sciences

For a reference copy of the document with all sections, see nature.com/documents/nr-reporting-summary-flat.pdf

Life sciences study design

All studies must disclose on these points even when the disclosure is negative.

Sample size	Samples sizes were determined to reveal statistical significance and reproducibility of the data. For some analyses sample size was limited by availability of appropriate materials (analysis of patient materials).
Data exclusions	No data were excluded.
Replication	DNase-seq and ChIP-sea analyses were performed at least twice. All other experiments were repeated at least three times in biological replicates. The exact number of repetitions is indicated in the figure legends. All replicates were successful; only clear technical failures were excluded.
Randomization	Randomization is indicated when applied. Patient samples for retrospective analyses were chosen based on availability.
Blinding	For the biochemical and cell biological experiments no blinding was performed due to the limited availability of persons performing the experiments.

Reporting for specific materials, systems and methods

We require information from authors about some types of materials, experimental systems and methods used in many studies. Here, indicate whether each material, system or method listed is relevant to your study. If you are not sure if a list item applies to your research, read the appropriate section before selecting a response.

Materials & experimental systems

n/a	Involved in the study	
☐	☒	Antibodies
☐	☒	Eukaryotic cell lines
☒	☐	Palaeontology and archaeology
☐	☒	Animals and other organisms
☒	☐	Clinical data
☒	☐	Dual use research of concern
☒	☐	Plants

Methods

n/a	Involved in the study	
☐	☒	ChIP-seq
☐	☒	Flow cytometry
☒	☐	MRI-based neuroimaging

Antibodies

Antibodies used	Antibodies used for EMSA (for dilution see Methods section / Supplementary table 7) :<br>anti-IRF4 (M-17), sc-6059, Santa Cruz Biotechnology<br>anti-JUNB (N-17), sc-46, Santa Cruz Biotechnology<br>anti-BATF (clone WW-8), sc-100974, Santa Cruz Biotechnology<br>anti-HA-tag (clone C29F4), #3724, Cell Signaling Technology<br>anti-FLAG (clone M2), #F1804, Sigma-Aldrich<br>anti-BATF (clone D7C5), #8638, Cell Signaling Technology<br>IgG1 mouse, MAB002, R&D Systems<br>IgG rabbit, AB-105-c, R&D Systems<br>IgG goat, AB-108-c, R&D Systems<br><br>Antibodies used for Western Blot (for dilution see Methods section / Supplementary table 7):<br>anti-IRF4 (M-17), sc-6059, Santa Cruz Biotechnology

anti-IRF4 (clone D9P5H), #15106, Cell Signaling Technology
anti-HA probe (Y-11), sc-805, Santa Cruz Biotechnology
anti-HA-tag (clone C29F4), #3724, Cell Signaling Technology
anti-FLAG (clone M2), F1804, Sigma-Aldrich
anti-BATF (clone D7C5), #8638, Cell Signaling Technology
anti-BATF (clone WW8), sc-100974, Santa Cruz Biotechnology
anti-c-Jun (H-79), sc-1694, Santa Cruz Biotechnology
anti-SNFT/BATF3 (M-13), sc-162246X, Santa Cruz Biotechnology
anti-PARP1 (clone F-2), sc-8007, Santa Cruz Biotechnology
anti-b-actin (clone AC-74), #A5316, Sigma-Aldrich

Antibodies used for flow cytometry (for dilution see Methods section / Supplementary table 7):
CD138-PE, #142504, BioLegend
PerCP/cy5.5 Anti-mouse B220, #103235, BioLegend

Antibody used for immunohistochemistry:
anti-IRF4 (clone MUM1p), #M725929, DAKO/Agilent.

Validation

Antibodies were validated as follows:

PARP1 sc-8007 antibody has been validated by the manufacturer including siRNA-mediated PARP1 knock-down [https://www.scbt.com/de/p/parp-1-antibody-f-2].

JUNB (N-17) antibody has been validated by immunoblotting of cell lines with validated JUNB expression or a lack thereof (Schleussner et al., Leukemia, 2018, 32:1994) in addition to the validation provided by the manufacturer [https://datasheets.scbt.com/sc-46.pdf].

BATF (WW8) antibody has been validated by immunoblotting following ectopic expression of BATF (our MS, see e.g. Supplementary fig. 3a) in addition to the informations provided by the manufacturer [https://www.scbt.com/de/p/b-atf-antibody-ww8].

BATF (D7C5) antibody has been validated by immunoblotting following ectopic expression of BATF (our MS, see e.g. Supplementary fig. 3f), analyses of cell lines with specific BATF-KO (Schleussner et al., Leukemia, 2018, 32:1994) as well as analyses of cell lines with validated BATF expression or a lack thereof (Schleussner et al., Leukemia, 2018, 32:1994) in addition to the informations provided by the manufacturer [https://www.cellsignal.com/products/primary-antibodies/batf-d7c5-rabbit-mab/8638].

c-JUN (H-79) antibody has been validated by immunoblotting following ectopic expression of c-JUN (our MS, see Supplementary fig. 3f) in addition to the informations provided by the manufacturer [https://datasheets.scbt.com/sc-1694.pdf].

SNFT/BATF3 (M-13) antibody has been validated by immunoblotting following ectopic expression of SNFT/BATF3 (our MS, Supplementary fig. 12c) in addition to the data provided by the manufacturer [https://datasheets.scbt.com/sc-162246.pdf].

IRF4 (M-17) antibody has been validated by immunoblotting following ectopic expression of IRF4 (our MS, e.g. Supplementary fig. 2a), analyses of cell lines with validated IRF4 expression or lack thereof (our MS, Supplementary fig. 1c), in addition to the informations provided by the manufacturer [https://datasheets.scbt.com/sc-6059.pdf].

IRF4 (D9PH5) antibody has been validated by immunoblotting following ectopic expression of IRF4 (our MS, Supplementary fig. 3f), in addition to the informations provided by the manufacturer [https://www.cellsignal.com/products/primary-antibodies/irf-4-d9p5h-rabbit-mab/15106].

b-actin (#5316) antibody. Informations on antibody validation are provided by the manufacturer [https://www.sigmaaldrich.com/deepweb/assets/sigmaaldrich/product/documents/335/840/a5316dat.pdf].

FLAG M2 (F1804) antibody has been validated following ectopic expression of FLAG-tagged proteins (e.g. JUNB, our MS, Supplementary fig. 3a) in addition to the informations provided by the manufacturer [https://www.sigmaaldrich.com/deepweb/assets/sigmaaldrich/product/documents/119/160/f1804bul-mk.pdf].

HA-tag (C29F4) antibody has been validated following ectopic expression of HA-tagged proteins (e.g. IRF4, our MS, e.g. Supplementary fig. 11a) in addition to the informations provided by the manufacturer [https://www.cellsignal.com/datasheet.jsp?productid=3724&images=1&size=A4].

HA-probe (Y-11) antibody has been validated following ectopic expression of HA-tagged proteins (e.g. IRF4, our MS, e.g. Supplementary fig. 2a) in addition to the informations provided by the manufacturer [https://datasheets.scbt.com/sc-805.pdf].

CD138-PE (142504) antibody. Informations on antibody validation are provided by the manufacturer [https://www.biolegend.com/de-at/products/pe-anti-mouse-cd138-syndecan-1-antibody-7519?GroupID=BLG9623].

PerCP/cy5.5 Anti-mouse B220 (103235) antibody. Informations on antibody validation are provided by the manufacturer [https://www.biolegend.com/de-at/products/percp-cyanine5-5-anti-mouse-human-cd45r-b220-antibody-4267].

Eukaryotic cell lines

Policy information about cell lines and Sex and Gender in Research

Cell line source(s)	Cell lines were obtained from the German Collection of Microorganisms and Cell Cultures (DSMZ), the American Type Culture Collection (ATCC) and other investigators (L591 from V. Diehl; L540Cy from A. Engert, both Cologne, Germany).
Authentication	Authenticity of all the cell lines was verified by STR fingerprinting.
Mycoplasma contamination	As a standard procedure in our laboratory all cell lines are regularly tested negative for mycoplasma contamination.
Commonly misidentified lines (See ICLAC register)	None of those have been used.

Animals and other research organisms

Policy information about studies involving animals; ARRIVE guidelines recommended for reporting animal research, and Sex and Gender in Research

Laboratory animals	C57BL/6 mice, 8-12 weeks old and originally obtained from Jackson laboratories were used for preparation of mouse splenic B cells. Housing conditions for mice comprise a 12 h dark/light cycle at a humidity of 55 +/- 10% at 22 +/- 2 °C.
Wild animals	The study did not involve wild animals.
Reporting on sex	Information on sex has bot specifically been collected. The reason for this is provided in the sex and gender section of this document.
Field-collected samples	The study did not involve samples collected from the field.
Ethics oversight	Animal experiments were approved by the local authority Landesamt für Gesundheit und Soziales (LAGeSo; X9027/11).

Note that full information on the approval of the study protocol must also be provided in the manuscript.

Plants

Seed stocks	N/A
Novel plant genotypes	N/A
Authentication	N/A

ChIP-seq

Data deposition

☒ Confirm that both raw and final processed data have been deposited in a public database such as GEO.

☒ Confirm that you have deposited or provided access to graph files (e.g. BED files) for the called peaks.

Data access links
May remain private before publication.

https://www.ncbi.nlm.nih.gov/geo/query/acc.cgi?acc=GSE211443&token=ezwzumyixzivnsb

Files in database submission

Sample / read1 / read2
IRF4_KM-H2_1 / KM-H2_IRF4_rep1_R1.fastq.gz / KM-H2_IRF4_rep1_R2.fastq.gz
IRF4_KM-H2_2 / KM-H2-IRF4_rep2_R1.fastq.gz / KM-H2-IRF4_rep2_R2.fastq.gz
IRF4_L428_1 / IRF4_S5_L001_R1_001.fastq.gz / IRF4_S5_L002_R1_001.fastq.gz
IRF4_L428_2 / IRF4_S5_L003_R1_001.fastq.gz / IRF4_S5_L004_R1_001.fastq.gz
JunB_KM-H2_1 / JunB_KM-H2_R1.fastq.gz / JunB_KM-H2_R2.fastq.gz
JunB_KM-H2_2 / 983_19L003104_JunB_KM-H2_R1.fastq.gz / 983_19L003104_JunB_KM-H2_R2.fastq.gz
JunB_L428_1 / H447.L428JunB_CGATGT_L004_R1_001.fastq.gz
JunB_L428_2 / H447.L428JunB_CGATGT_L005_R1_001.fastq.gz

bigwig (bedGraph version) files:
GSE211443_KM-H2_IRF4_combined_hg19.bw
GSE211443_KM-H2_JunB_combined_hg19.bw
GSE211443_L428_IRF4_combined_hg19.bw
GSE211443_L428_JunB_combined_hg19.bw

Genome browser session (e.g. UCSC)

http://genome-euro.ucsc.edu/s/Dr%20Pierre%20Cauchy/HL_ChIP_DHS

Methodology

Replicates

Replicates were done in duplicates and were grown in separate flasks. The ChIP reaction, library preparation and sequencing were performed per replicate independently.

Sequencing depth

sample / total_reads / aligned_reads / type / read_length
IRF4_KM-H2_1 / 83965394 / 80849432 / paired-end / 75
IRF4_KM-H2_2 / 86991062 / 84182235 / paired-end / 75
IRF4_L428_1 / 71731067 / 71224300 / single-end / 75
IRF4_L428_2 / 71926680 / 71410347 / single-end / 75
JunB_KM-H2_1 / 29751820 / 27045023 / paired-end / 100
JunB_KM-H2_2 / 87028198 / 80470381 / paired-end / 100
JunB_L428_1 / 32240524 / 31585792 / single-end / 50
JunB_L428_2 / 26089648 / 25565663 / single-end / 50

Antibodies

IRF4: sc-6059-X, Santa Cruz Biotechnology
JUNB: sc-46-X, Santa Cruz Biotechnology

Peak calling parameters

macs2 (v2.1.0) callpeak -g hs -q 0.001 -B --SPMR --trackline
<trackline> --keep-dup auto

Data quality

Read qualities were checked with fastqc. 33082 (KM-H2 IRF4 combined), 30022 (L428 IRF4 combined), 24914 (KM-H2 JUNB combined) and 24886 (L428 JUNB) peaks were at FDR<0.1% (q 0.001). All were above 5-fold enrichment.

Software

HiSeq Analysis Software v2.0 (Illumina)
bcl2fastq v2.16.0 (Illumina)
bowtie2 v2.1.0
samtools v1.1
macs2 v2.1.0
bedtools v2.19.0
homer v4.6
ChIPpeakAnno v.1.12.0
heatmap.2 v2.17.0
pyBedtools 0.9.0

Flow Cytometry

Plots

Confirm that:

☒ The axis labels state the marker and fluorochrome used (e.g. CD4-FITC).

☒ The axis scales are clearly visible. Include numbers along axes only for bottom left plot of group (a 'group' is an analysis of identical markers).

☒ All plots are contour plots with outliers or pseudocolor plots.

☒ A numerical value for number of cells or percentage (with statistics) is provided.

Methodology

Sample preparation

Retrovirally transduced B cells were harvested, blocked with TruStain FcX (a-mouse CD16/32; 10 min, 4°C; #101320. BioLegend) and stained (20 min, 4°C) with B220-PerCP/Cyanine5.5 (#103235; BioLegend) and CD138-PE (#142504; BioLegend) in PBS, pH7.2, supplemented with 3% FCS and 1 mM EDTA.

Instrument

FACSCantoII

Software

FlowJo software (BD FlowJo, PRID:SCR_008520; v9.9.6) was used to generate plots.

Cell population abundance

Cell population abundances are indicated in the respective plots.

Gating strategy

FSC/SSC gating was performed to exclude debris and define living cells. Next, within the viable cells GFP+ cells were gated (SSC/GFP). Finally, the percentage of CD138high/B220low cells within the GFP+ cells was determined. The gating strategy is to be deduced from the main figure part provided, and thus there is no need for a further Supplementary figure.

☐ Tick this box to confirm that a figure exemplifying the gating strategy is provided in the Supplementary Information.
