# Supplementary material for: Transcriptional reprogramming by mutated IRF4 in lymphoma
Source: Nat Commun. 2023 Nov 7;14:6947. doi: 10.1038/s41467-023-41954-8 (PMC10630337; doi:10.1038/s41467-023-41954-8)
Supplement: Supplementary file 4 — Description of Additional Supplementary Files [file 41467_2023_41954_MOESM4_ESM.docx]

**Description of Additional Supplementary Files**

Supplementary Data 1

Description: Differentially expressed genes in IRF4-WT and IRF4-C99R BJAB cells.

Supplementary Data 2

Description: Differentially expressed genes in C57BL/6 splenic B cells transduced with IRF4 variants.
